# Antibiotics and extracorporeal circulation – one size does not fit all

**DOI:** 10.1186/s13054-014-0695-6

**Published:** 2014-12-19

**Authors:** João Gonçalves-Pereira, Bruno Oliveira

**Affiliations:** Intensive Care Unit, Vila Franca de Xira Hospital, Rua Dr. Luis Cesar Pereira 1, 2600-178 Vila Franca de Xira, Portugal; Faculdade Ciências Médicas, Universidade Nova de Lisboa, Campo dos Mártires da Pátria, 130, Lisboa, 1169-056 Portugal

## Abstract

Dosing of antibiotics in critically ill patients is a significant challenge. The increasing number of patients undergoing extracorporeal membrane oxygenation further complicates the issue due to inflammatory activation and to drug sequestration in the circuit. Since patients receiving extracorporeal membrane oxygenation commonly face severe infections, appropriate antibiotic selection and correct dosing is of paramount importance to improve survival. Therapeutic drug monitoring (whenever available) or population pharmacokinetics, based on readily available clinical and laboratory data, should help tailor antibiotic dosing to the individual patient.

In a recent edition of *Critical Care*, on behalf of the Antibiotic, Sedative and Analgesic Pharmacokinetics during Extracorporeal Membrane Oxygenation study investigators, Shekar and colleagues assessed meropenem pharmacokinetics (PK) in patients receiving extracorporeal membrane oxygenation (ECMO), with or without continuous renal replacement therapy (CRRT) [1].

Patients on ECMO commonly have infections, reaching rates of more than 15 per 1,000 ECMO days according to data pooled from the Extracorporeal Life Support Organization Registry [2], and these infections are associated with increased mortality. There is also increasing evidence that initial antibiotic appropriateness impacts the outcome of severe infections. To ensure maximal bacterial killing, antibiotics should achieve adequate exposure as soon as possible; that is, an adequate concentration during an ideal time, according to the antibiotic’s characteristics. Consequently, not only starting an antibiotic but also selecting an ideal dose is crucial for therapeutic success.

High antibiotic doses are usually needed to achieve therapeutic concentrations early in the infection course. This has been shown for β-lactams [3], vancomycin [4] and aminoglycosides [5], implying the need for higher than recommended doses. These high doses may also be necessary to overcome the impact of bacteria resistance, especially for hospital-acquired infections.

Antibiotics are usually prescribed in a traditional pattern, taking into account only the existence of renal or liver dysfunction and considering the susceptibility pattern of the microorganism. Moreover, the antibiotic dose is usually maintained throughout the treatment course, although significant PK changes occur from the resuscitation phase to the recovery phase [6].

Ideally, individualized dosing strategies should account for the altered PK and pathogen susceptibility in each patient. In order to achieve this goal it is necessary to understand the full impact of therapeutic interventions and patient characteristics on antibiotic PK.

Drug dosing in critically ill patients is especially challenging due to the frequent and hard to predict changes in PK, especially the increased volume of distribution and clearance variation [7], secondary to volume resuscitation, increased cardiac output and capillary leak. Several therapeutic procedures are also associated with PK changes, and both CRRT and ECMO are among the most challenging. In fact the inflammatory activation induced by the extracorporeal circulation and exposure of blood to foreign material and the drug sequestration in the circuit [8] both contribute to alteration in antibiotic concentration and half-life.

ECMO is among the most rapidly increasing support techniques in intensive care [9], and its use in the United States has increased more than four times in a space of only 5 years. Understanding the technique’s impact on antibiotic PK is therefore essential.

In their study, Shekar and colleagues showed that the volume of distribution of meropenem is increased in patients undergoing ECMO (with or without CRRT) and consequently high initial antibiotic doses were commonly needed [1]. On the other hand, clearance was usually low, correlated with CRRT and with creatinine clearance, so subsequent doses had to be adjusted. Although conventional dosing was able to achieve a trough concentration >2 mg/dl in all patients, a higher target (>8 mg/dl) was only obtained when higher doses were used [1]. These conclusions were in line with another recently published study that accessed patients undergoing CRRT [10]: conventional dosing was not able to achieve the intended target concentrations in all patients, but increasing doses also exposed several patients to toxicity.

Therapeutic drug monitoring has been shown to facilitate the achievement of adequate antibiotic concentration [11]. Unfortunately this treatment is only routinely available for vancomycin and aminoglycosides. Studies addressing β-lactam antibiotics PK, however, have also unveiled underdosing and toxic accumulation [12] due to large interindividual and intraindividual variability, suggesting the need to tailor the antibiotic dose to the patient and to adjust it according to PK changes [13,14].

The study by Shekar and colleagues is part of a larger multicentric effort aimed at the determination of PK of multiple drugs, namely sedatives, opioids, antibiotics and antifungals, in ECMO patients [15]. This study is a painstaking work of undeniable importance that will culminate in the determination of evidence-based guidelines for drug therapy dosage during ECMO.

In conclusion, we can clearly no longer rely on the ‘one size fits all’ paradigm when choosing the antibiotic dose. Knowledge of antibiotic PK, patient status and immune function, bacteria virulence, susceptibility and inoculum, as well as the PK impact of different therapies, should all contribute to dose selection.

## Abbreviations

CRRT: Continuous renal replacement therapy
ECMO: Extracorporeal membrane oxygenation
PK: Pharmacokinetics

## References

[1] Shekar K, Fraser JF, Taccone FS, Welch S, Wallis SC, Mullany DV, Lipman J, Roberts JA (2014). The combined effects of extracorporeal membrane oxygenation and renal replacement therapy on meropenem pharmacokinetics: a matched cohort study. Critical Care.

[2] Bizzarro MJ, Conrad SA, Kaufman DA, Rycus P (2011). Infections acquired during extracorporeal membrane oxygenation in neonates, children, and adults. Pediatr Crit Care Med.

[3] Taccone FS, Laterre PF, Dugernier T, Spapen H, Delattre I, Witebolle X, De Backer D, Layeux B, Wallemacq P, Vincent JL, Jacobs F (2010). Insufficient beta-lactam concentrations in the early phase of severe sepsis and septic shock. Crit Care.

[4] Baptista JP, Sousa E, Martins PJ, Pimentel JM (2012). Augmented renal clearance in septic patients and implications for vancomycin optimisation. Int J Antimicrob Agents.

[5] Goncalves-Pereira J, Martins A, Povoa P (2010). Pharmacokinetics of gentamicin in critically ill patients: pilot study evaluating the first dose. Clin Microbiol Infect.

[6] Triginer C, Izquierdo I, Fernandez R, Rello J, Torrent J, Benito S, Net A (1990). Gentamicin volume of distribution in critically ill septic patients. Intensive Care Med.

[7] Goncalves-Pereira J, Povoa P (2011). Antibiotics in critically ill patients: a systematic review of the pharmacokinetics of beta-lactams. Crit Care.

[8] Shekar K, Roberts JA, McDonald CI, Fisquet S, Barnett AG, Mullany DV, Ghassabian S, Wallis SC, Fung YL, Smith MT, Fraser JF (2012). Sequestration of drugs in the circuit may lead to therapeutic failure during extracorporeal membrane oxygenation. Crit Care.

[9] Sauer CM, Yuh DD, Bonde P: **Extracorporeal membrane oxygenation (ECMO) use has increased by 433% in adults in the United States from 2006 to 2011.** *ASAIO J* 2014. [Epub ahead of print] 10.1097/MAT.0000000000000160 25303799

[10] Beumier M, Casu GS, Hites M, Seyler L, Cotton F, Vincent JL, Jacobs F, Taccone FS (2014). Beta-lactam antibiotic concentrations during continuous renal replacement therapy. Crit Care.

[11] Pea F, Bertolissi M, Di Silvestre A, Poz D, Giordano F, Furlanut M (2002). TDM coupled with Bayesian forecasting should be considered an invaluable tool for optimizing vancomycin daily exposure in unstable critically ill patients. Int J Antimicrob Agents.

[12] Roberts JA, Ulldemolins M, Roberts MS, McWhinney B, Ungerer J, Paterson DL, Lipman J (2010). Therapeutic drug monitoring of beta-lactams in critically ill patients: proof of concept. Int J Antimicrob Agents.

[13] Goncalves-Pereira J, Paiva JA (2013). Dose modulation: a new concept of antibiotic therapy in the critically ill patient?. J Crit Care.

[14] De Waele JJ, Lipman J, Akova M, Bassetti M, Dimopoulos G, Kaukonen M, Koulenti D, Martin C, Montravers P, Rello J, Rhodes A, Udy AA, Starr T, Wallis SC, Roberts JA (2014). Risk factors for target non-attainment during empirical treatment with beta-lactam antibiotics in critically ill patients. Intensive Care Med.

[15] Shekar K, Roberts JA, Welch S, Buscher H, Rudham S, Burrows F, Ghassabian S, Wallis SC, Levkovich B, Pellegrino V, McGuinness S, Parke R, Gilder E, Barnett AG, Walsham J, Mullany DV, Fung YL, Smith MT, Fraser JF (2012). ASAP ECMO: Antibiotic, Sedative and Analgesic Pharmacokinetics during Extracorporeal Membrane Oxygenation: a multi-centre study to optimise drug therapy during ECMO. BMC Anesthesiol.

